# Comparison of Inflation and Ventilation with Hydrogen Sulfide during the Warm Ischemia Phase on Ischemia-Reperfusion Injury in a Rat Model of Non-Heart-Beating Donor Lung Transplantation

**DOI:** 10.1155/2023/3645304

**Published:** 2023-02-02

**Authors:** Junwei Yan, Bing Zhang, Junmin Yu, Xuan Wang, Han Zhao, Guangqi Li, Yanwei Yin, Chao Meng

**Affiliations:** ^1^Department of Vascular Surgery, The Affiliated Hospital of Qingdao University, Qingdao, China 266000; ^2^Department of Anesthesiology, The Affiliated Hospital of Qingdao University, Qingdao, China 266000; ^3^Department of Pain Management, The Affiliated Hospital of Qingdao University, Qingdao, China 266000; ^4^Department of Pathology, The Affiliated Hospital of Qingdao University, Qingdao, China 266000

## Abstract

Donor lung ventilation and inflation during the warm ischemia could attenuate ischemia-reperfusion injury (IRI) after lung transplantation. Hydrogen sulfide (H_2_S), as a kind of protective gas, has demonstrated the antilung IRI effect. This study is aimed at observing the different methods of administering H_2_S in the setting of warm ischemia, ventilation, and inflation on the lung graft from a rat non-heart-beating donor. After 1 h of cardiac arrest, donor lungs in situ were inflated with 80 ppm H_2_S (FS group), ventilated with 80 ppm H_2_S (VS group), or deflated (control group) for 2 h. Then, the lung transplantation was performed after 3 h cold ischemia. The rats without ischemia and reperfusion were in the sham group. Pulmonary surfactant in the bronchoalveolar lavage fluid was measured in donor lung. The inflammatory response, cell apoptosis, and lung graft function were assessed at 3 h after reperfusion. The lung injury was exacerbated in the control group, which was attenuated significantly after the H_2_S treatment. Compared with the FS group, the pulmonary surfactant in the donor was deteriorated, the lung oxygenation function was decreased, and the inflammatory response and cell apoptosis were increased in the graft in the VS group (*P* < 0.05). In conclusion, H_2_S inflation during the warm ischemia phase improved the function of lung graft via regulating pulmonary surfactant stability and decreased the lung graft IRI via decreasing the inflammatory response and cell apoptosis.

## 1. Introduction

Currently, the shortage of suitable donor has become the main limiting factor to lung transplantation (LTx) [1]. With the increasing demand of donor, more attention is paid on non-heart-beating donors (NHBDs). However, warm ischemia of donor lung, the special stage of NHBDs, makes the donor injury, decreases the donor quality, and aggravates early primary graft dysfunction (PGD) and ischemia-reperfusion injury (IRI) after LTx [2]. Thus, the donor lung from NHBDs needs in situ conservation immediately [3].

Hydrogen sulfide (H_2_S) was appreciated as a new member of important cellular signaling molecule characterized by metabolism inhibition [4]. H_2_S inhalation has been reported to mitigate lung injury induced by sepsis, lipopolysaccharide (LPS), and burn [5, 6, 7]. Ventilation with 50% oxygen (O_2_) in situ during the warm ischemia could also enhance the function of lung graft [8]. Our previous study showed that H_2_S inflation in vitro lung tissues in the setting of warm ischemia phase attenuated lung IRI in a model of rat LTx via regulating metabolism [9]. Therefore, the aim of this study was to compare the effects of different methods of administering H_2_S in the setting of warm ischemia, ventilation, and inflation on donor lungs and IRI of lung grafts from NHBDs in rats.

## 2. Methods and Materials

### 2.1. Animals

Adult pathogen-free inbred male SD rats (Vital River Laboratories, Beijing, China) weighting 250-300 g were housed in the lab in a 12 h-12 h light-dark cycle for one week before the experiment. This study was approved by the Animal Care and Welfare Committee of the Affiliated Hospital of Qingdao University.

### 2.2. Non-Heart-Beating Donor Model and Groups

**Figure 1:**
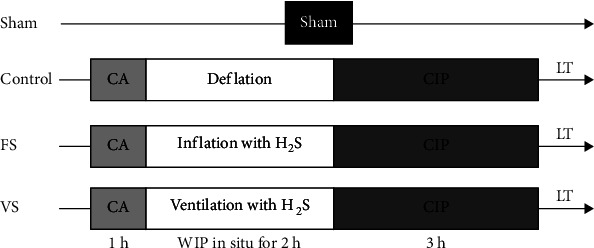
Study design. CA: cardiac arrest; CIP: cold ischemia phase; WIP: warm ischemia phase; LT: lung transplantation; FS: H_2_S inflation group; VS: H_2_S ventilation group.

Donor rats were anesthetized with intraperitoneal injection of sodium pentobarbital (60 mg/kg) and intubated via tracheostomy. After heparinization (200 U/kg), rats were exsanguinated via the abdominal aorta until cardiac arrest. Following 1 h “hand-off” period, 3 groups were randomized (*n* = 8). In the control group, the donor lung was deflated without any treatment. In the H_2_S inflation group (FS group), the donor lung was inflated with 80 parts per million (ppm) H_2_S in 40% O_2_+nitrogen (N_2_) (7 ml/kg) which were replaced every 20 min using an airtight injector (Agilent Technologies Corporation, California, USA). In the H_2_S ventilation group (VS group), the donor lung was ventilated with the same mixed gas (7 ml/kg) at 10 rates/min with a positive end-expiratory pressure (PEEP) of 5 cm H_2_O. After 2 h of treatment, the donor lung was flushed with 20 ml 4°C low-potassium dextran (LPD) solution from the pulmonary artery with a 20 cm H_2_O pressure for 10 min and preserved in 4°C LPD solution for another 3 h. Then, the LTx was performed, and the recipients were euthanized via exsanguination after reperfusion for 3 h. Additionally, in the sham group, thoracotomies were performed on rats but no LTx, and the following procedure was similar to the recipients (Figure 1).

### 2.3. Lung Transplantation

Orthotopic left LTx was performed using a modified cuff technique [10]. After anesthesia, the femoral artery of recipients was cannulated for monitoring blood pressure (AS/3, Datex, Helsinki, Finland). Recipients were intubated and ventilated (10 ml/kg) with 40% O_2_+60% N_2_ and a PEEP of 5 cm H_2_O. The arterial carbon dioxide tension (PaCO_2_) was maintained in 35-45 mmHg via regulating respiratory frequency. During the experiment, the rats were given sodium pentobarbital and pipecuronium bromide for anesthesia maintenance.

### 2.4. Detection of Phospholipid and Pulmonary Surfactant Protein

After the ischemia phase, the 10 ml cold normal saline was injected into the donor lung through the trachea for 3 times. The saline in each time was infused and withdrawn slowly for 3 times [11]. The collected saline, bronchoalveolar lavage fluid (BALF), was centrifuged at 150 g for 10 min. The supernatant was used to measure the phospholipid via phosphorus measurement using a Phosphate Assay kit (Jiancheng Biotechnology, Nanjing, China), and the resulting pellet was obtained to measure the large surfactant aggregates (LA) and small surfactant aggregates (SA) through centrifuging at 40000 g for 15 min.

### 2.5. Blood Gas Analysis

During the experiment, the arterial blood gas analyses were performed in recipients at T0-T4 time points which meant baseline (3 min after ventilation), 3 min, 1 h, 2 h, and 3 h after reperfusion, correspondingly. At the end of the experiment, the blood collection in the left pulmonary vein was also performed for blood gas analysis (Rapidlab 248, Bayer, Medfield, USA).

### 2.6. Detection of Inflammatory Indices

After 3 h of reperfusion, the levels of interleukin- (IL-) 6, IL-10, and tumor necrosis factor- (TNF-) *α* in serum were detected using enzyme-linked immunosorbent assay kits (R&D Systems, MN, USA). The wet-to-dry weight (W/D) ratio was detected in the upper-lobe left lung graft by desiccating at 80°C for 72 h. The lower-lobe left lung graft was homogenized with cold normal saline to detect the myeloperoxidase (MPO) activity using a Regent-Box (Jiancheng Biotechnology, Nanjing, China).

### 2.7. Detection of Histopathology

After 3 h of reperfusion, the middle-lobe left lung graft was fixed in 4% paraformaldehyde for 36 h and then embedded in paraffin and cut into 6 *μ*m thick sections. Following the staining with hematoxylin and eosin, the histological analysis was performed based on the following criteria: neutrophil infiltration, airway epithelial cell damage, edema, hyaline membrane formation, and hemorrhage. Each criterion has 5 grade changes: normal, minimal, mild, moderate, and severe change, which was recorded as 0-4, correspondingly [12]. All sections were examined by a blinded pathologist.

### 2.8. Detection of Cell Apoptosis

After 3 h of reperfusion, the alveolar epithelial cellular apoptosis was detected through the terminal deoxynucleotidyl transferase dUTP nick end labeling (TUNEL) staining by kit (Zhongshan Golden Bridge Biotechnology, Beijing, China). The number of positive cells in every 100 cells in five random high-power (×40) fields in the same section was recorded as apoptotic index (AI) [13]. Immunohistochemical staining was used to observe the level of caspase-3 protein expression in the alveolar epithelial cells by a special assay (Zhongshan Golden Bridge Biotechnology, Beijing, China). Immunohistochemical scores (IHS) of caspase-3 we evaluated in five random high-power (×40) fields in every specimen, which were determined by multiplying the quantity score by the staining intensity score. Quantity scores, an estimation of the percentage of immunoreactive cells, were graded in 5: no staining, 1%-10% of cells staining, 11%-50% of cells staining, 51%-80% of cell staining, and 81%-100% of cells staining, which were recorded as 0-4, correspondingly. Staining intensity, an estimation of the staining intensity, was rated on a scale of 0-3, which meant negative, weak, moderate, and strong, correspondingly [14]. All sections were examined by a blinded pathologist.

### 2.9. Lung Static Compliance

The static lung compliance was evaluated after the recipients were euthanized via exsanguination. Median sternotomy was performed, and the lung was used to measure the pressure-volume (P-V) curves by an apparatus to determine the static lung compliance. Airway pressure was changed in a stepwise interval of 5 cm H_2_O to 30 cm H_2_O. Lung volumes were recorded following a 1 min stabilization [13].

### 2.10. Statistical Analysis

Data were expressed as the mean values ± standard deviations for quantitative data. Differences in groups were analyzed by one-way analysis of variance (ANOVA) and the Student-Newman-Keuls test using SPSS 20.0. Repeated data were analyzed by two-way ANOVA followed by Dunnett's test. The nonparametric method with the Kruskal-Wallis test was used for the analysis of LIS data. A *P* value less than 0.05 was considered to be statistically significant.

## 3. Results

### 3.1. Basic Data

All rats used in this study had no statistical difference in basic conditions. The control group and the FS group had one recipient excluded for the failure of the lung hilum separation and hemodynamic instability. Eventually, 8 pairs of rats were included in each group and 8 rats in the sham group. The experiment-related relative time, including warm ischemia, cold ischemia, and transplantation time, in each group had no statistical differences (Table 1).

### 3.2. Phospholipids and Pulmonary Surfactant Protein in BALF of Donor Lung

**Figure 2:**
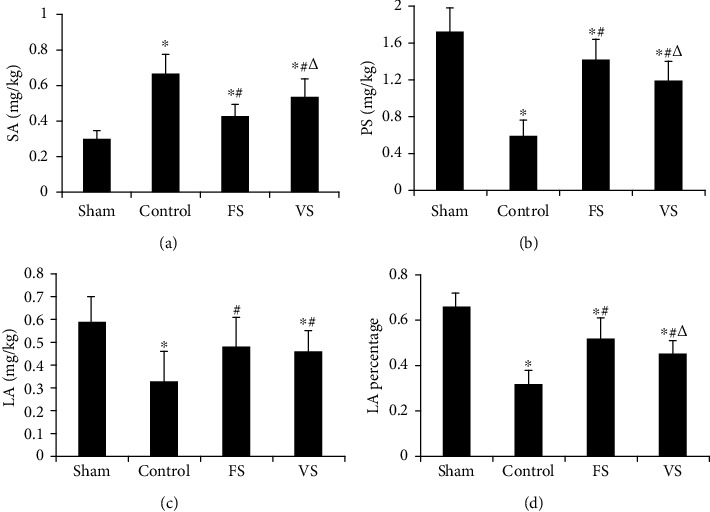
Pulmonary surfactant detection in BALF (*n* = 8). During the warm ischemia phase, when the treatment stopped, the BALF in each group was used to detect the different composition of pulmonary surfactant. BALF were directly collected from rats in the sham group. (a) SA content, (b) PS content, (c) LA content, and (d) LA percentage. SA: small surfactant aggregates; LA: large surfactant aggregates; PS: phospholipids; BALF: bronchoalveolar lavage fluid; FS: H_2_S inflation; VS: H_2_S ventilation. ^∗^*P* < 0.05 vs. sham group; ^#^*P* < 0.05 vs. control group; ^△^*P* < 0.05 vs. FS group.

The phospholipid content, LA content, and LA percentage in BALF decreased, and SA content in the control group was higher than those in the sham group (*P* < 0.05). Compared with the control group, the phospholipid content, LA content, and LA percentage increased and SA content decreased significantly in the FS group (*P* < 0.05). However, these indices in the VS group were higher than those in the FS group (*P* < 0.05) (Figure 2). LA content in the FS and VS groups showed no significant difference.

### 3.3. Oxygenation in Lung Graft

Rats in the sham group showed stable blood gas analysis indices. At 3 h after reperfusion, the oxygenation index, calculated through the partial pressure of arterial oxygen (PaO_2_)/fraction of inspired oxygen (FiO_2_), was significantly lower in the control group (267 ± 51 mm Hg) compared with the sham group (443 ± 19 mm Hg), and the oxygenation index in the FS group (376 ± 27 mm Hg) and the VS group (335 ± 36 mm Hg) was higher than the control group (*P* < 0.05). Additionally, compared with the FS group, this index was lower in the VS group (*P* < 0.05). The base excess (BE) and pH values showed a similar tendency as oxygenation index (Table 2). Moreover, the blood gas analysis results of left pulmonary vein displayed a similar tendency as those showed above (Table 3).

### 3.4. Inflammatory Response in Recipients

Compared with the sham group (4.5 ± 0.6), the W/D ratio in the control group (8.3 ± 1.4) was decreased and that in the FS group (5.0 ± 0.8) and the VS group (5.7 ± 1.1) was lower than the control group (*P* < 0.05). However, the W/D ratio in the FS group and the VS group had no significant difference. Compared with the sham group, the levels of IL-6 and TNF-*α* in serum were increased in the control group (*P* < 0.05). And those in the FS group decreased compared with the control group, and the VS group showed an increase in IL-6 and TNF-*α* compared with the FS group (*P* < 0.05). The MPO activity, an index of the infiltration of neutrophils, showed a similar tendency as IL-6, and the serum level of anti-inflammatory factor, IL-10, exhibited a converse tendency as IL-6 (Table 4).

### 3.5. Lung Injury Score of Lung Graft

**Figure 3:**
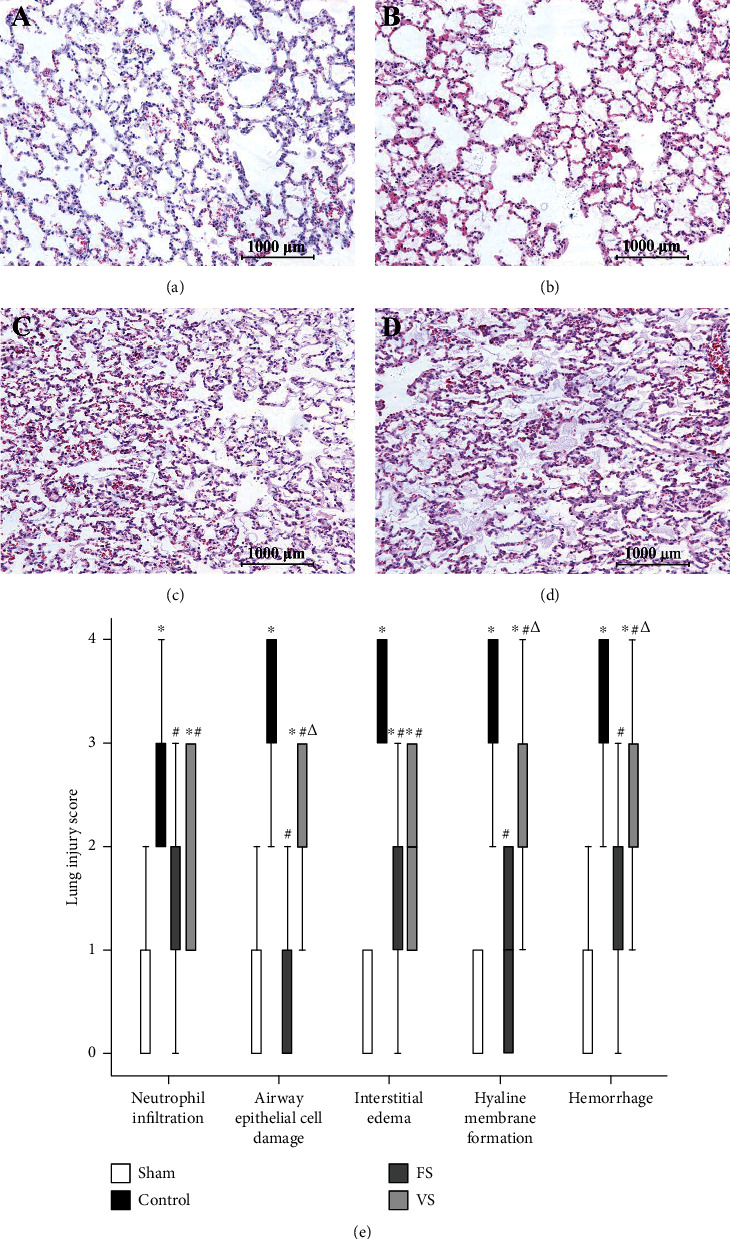
Histological analyses of the lung tissues (original magnification, 10x). In the sham group (a), lung tissues showed normal structure, while many abnormalities were showed in the control group (b), including severe interstitial edema, much neutrophil infiltration, and hemorrhage. After H_2_S treatment, smaller changes in the grafts were observed, and compared with the FS group (c), few edema and less hemorrhage were found in the VS group (d). (e) lung injury score (*n* = 5). FS: H_2_S inflation; VS: H_2_S ventilation. ^∗^*P* < 0.05 vs. sham group; ^#^*P* < 0.05 vs. control group; ^△^*P* < 0.05 vs. FS group.

Lung tissue in the sham group showed normal structure. Severe edema and hemorrhage were found in the control group. However, few changes could be observed in the FS group and the VS group. Therefore, compared with the sham group (0.5 (0 to 1)), the LIS of edema in the control group (3.5 (3 to 4)) increased significantly (*P* < 0.05), which in the FS group (1.5 (0 to 3)) and the VS group (2 (1 to 3)) was lower than the control group (*P* < 0.05). Compared with the FS group, the LIS of edema in the VS group increased significantly (*P* < 0.05). Additionally, similar tendencies were shown for the other criteria of LIS (Figure 3).

### 3.6. Cell Apoptosis in Lung Graft

**Figure 4:**
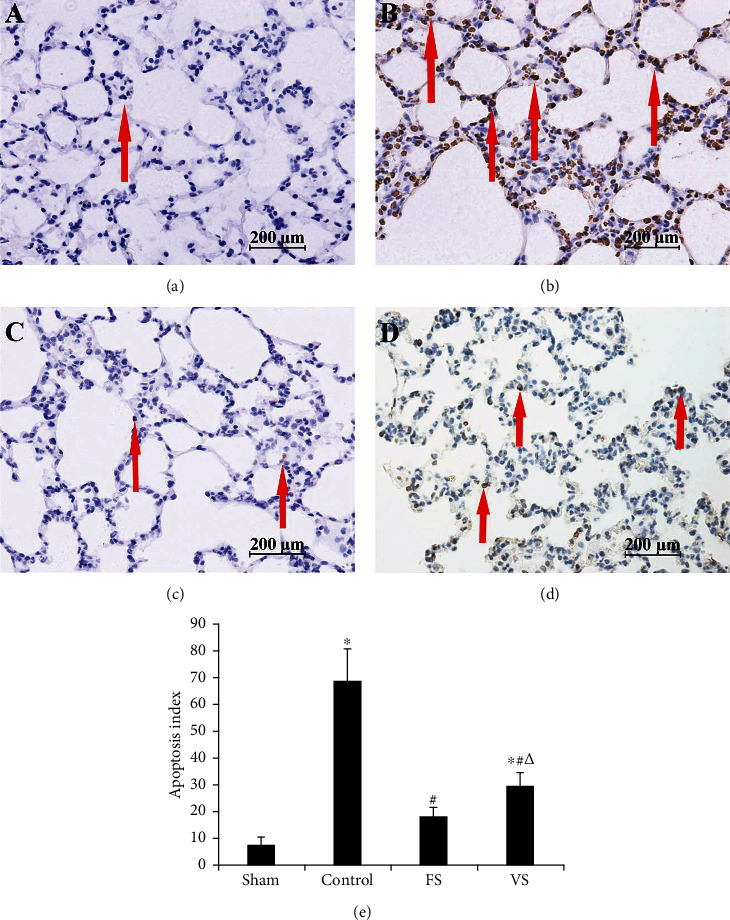
Cell apoptosis was measured by TUNEL staining (original magnification, 40x). The brown nuclear staining cells were recognized as the positive cells (as arrows showed). There were almost no TUNEL positive cells in the sham group (a), while too many in the control group (b). H_2_S treatment could decrease the number of TUNEL positive cells significantly, and less positive cells were noted in the FS group (c) compared with the VS group (d). (e) apoptosis index (*n* = 5). Data were showed as mean ± SD. TUNEL: terminal deoxynucleotidyl transferase dUTP nick end labeling; FS: H_2_S inflation; VS: H_2_S ventilation. ^∗^*P* < 0.05 vs. sham group; ^#^*P* < 0.05 vs. control group; ^△^*P* < 0.05 vs. FS group.

**Figure 5:**
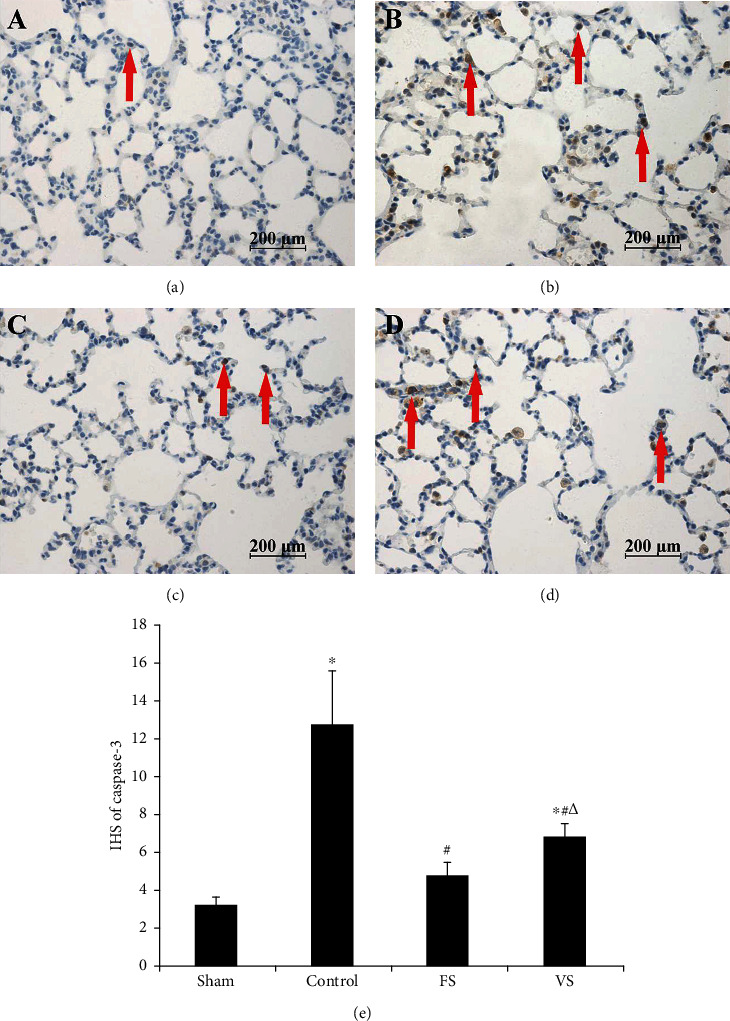
Expression of caspase-3 by immunohistochemistry (original magnification, 40x). The positive cell was represented as brown granules in the cytoplasm (as arrows showed). The sham group (a) showed almost no positive cells, while the control group (b) showed numerous positive cells. However, H_2_S treatment decreased the positive cells, and fewer were found in the FS group (c) compared with the VS group (d). (e) IHS of caspase-3 (*n* = 5). Data were showed as mean ± SD. IHS: immunohistochemical score; FS: H_2_S inflation; VS: H_2_S ventilation. ^∗^*P* < 0.05 vs. sham group; ^#^*P* < 0.05 vs. control group; ^△^*P* < 0.05 vs. FS group.

In the sham group, there were few TUNEL positive cells, while in the control group, large amounts of TUNEL positive cells were found. However, less TUNEL positive cells were showed in the FS and VS groups (Figure 4). Therefore, the AI in the control group was 48.8 ± 10.9, which was significantly higher than 5.4 ± 2.9 in the sham group (*P* < 0.05). And the AI in the FS group (19.6 ± 6.3) and the VS group (9.4 ± 2.3) was lower than the control group, while the AI in the VS group was higher than the FS group (*P* < 0.05). Additionally, the IHS of caspase-3 displayed a similar result as AI (Figure 5).

### 3.7. Static Compliance of Lung Graft

**Figure 6:**
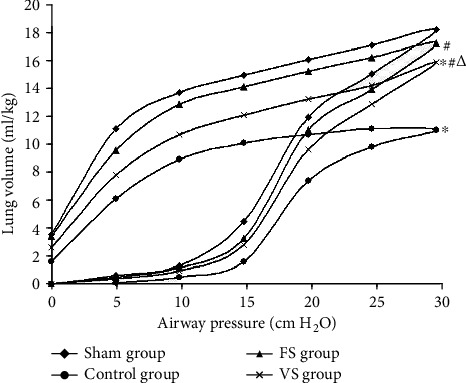
Lung static compliance. The lung static compliance was determined by the pressure-volume (P-V) curves (*n* = 5). Data were mean values, and the bars were omitted for clarity. FS: H_2_S inflation; VS: H_2_S ventilation. ^∗^*P* < 0.05 vs. sham group; ^#^*P* < 0.05 vs. control group; ^△^*P* < 0.05 vs. FS group.

Compared with the sham group, the P-V curve value decreased significantly in the control group, which was higher in the FS and VS groups compared with the control group (*P* < 0.05). However, that in the VS group decreased markedly compared with the FS group (*P* < 0.05). At a pressure of 30 cm H_2_O, the value in the sham was 18.5 ± 0.6 ml/kg, in the control group was 11.2 ± 1.0 ml/kg, in the FS group was 17.5 ± 1.1 ml/kg, and in the VS groups was 16.1 ± 0.5 ml/kg (Figure 6).

## 4. Discussion

This study demonstrated that H_2_S treatment during the warm ischemia phase improved donor lung quality via decreasing the SA content and increasing the LA percentage and the phospholipids content in donor lung. Furthermore, compared with ventilation, H_2_S inflation showed better protective effects on donor lung quality and decreased lung graft IRI via decreasing the inflammatory response and cell apoptosis.

Pulmonary surfactant, thought to consist of phospholipids and surfactant proteins, could decrease the surface tension of alveolar gas-liquid interface and maintain the pulmonary compliance. Phospholipids decreased the surface tension directly, and surfactant protein provided further adhesion on the alveolar surface and regulated the synthesis and secretion of phospholipids [15]. In addition, pulmonary surfactant proteins included two forms, the LA with biological activity and the SA without biological activity [16]. In this study, donor lung quality during the warm ischemia phase was decreased indicated by lower phospholipid content and the LA percentage. And H_2_S treatment improved donor lung quality via increasing the phospholipid content and the LA percentage and ameliorated lung compliance. Due to that, the phospholipid content, LA percentage, and P-V curves in the FS group were higher than the VS group, so H_2_S inflation provided better donor lung quality and graft compliance. Thus, H_2_S inflation during the warm ischemia phase protected against the donor lung injury via regulating the pulmonary surfactant better compared with ventilation.

Previous study has been demonstrated that the H_2_S could regulate pulmonary surfactant [17]. This effect may be associated with the following reasons. First, H_2_S may influence the synthesis and secretion of pulmonary surfactant. Second, H_2_S may induce chemical changes in the surfactant components [17].

In this study, H_2_S treatment during the warm ischemia phase decreased the systemic inflammatory response (e.g., IL-6 and TNF-*α*) and reduced the local inflammatory response (e.g., MPO content) in the recipients. This result was consistent with previous study. Faller et al. [18] reported that H_2_S treatment provided anti-inflammatory effects by reducing cytokine release and neutrophil transmigration in lung injury induced by ventilator. Wu et al. [19] found that exogenous H_2_S decreased the graft IL-*β* level and increased the graft IL-10 level in a model of rat experimental lung transplantation. Additionally, compared with ventilation, H_2_S inflation provided a more powerful role in inhibiting inflammatory response induced by ischemia reperfusion indicated by the less graft MPO content, less IL-6 and TNF-*α*, and more IL-10 in serum, which provided the evidence that inflation showed better effects on anti-inflammatory function.

Cell apoptosis also plays a key role in the IRI, which aggravated lung graft injury and decreased lung function [20]. Caspase-3 imparts an irreplaceable role in cell apoptosis and involved in the execution of apoptosis [21]. In this study, H_2_S treatment during the warm ischemia phase decreased the caspase-3 expression and apoptotic cell significantly after the reperfusion. H_2_S induced antiapoptotic effects were reported in ventilator-induced lung injury with the reduced TUNEL positive cells [18]. Liu et al. [22] found that exogenous H_2_S treatment decreased the TUNEL positive cells and inhibited the cell apoptosis via regulating the Fas protein expression in oleic acid-induced acute lung injury. Furthermore, H_2_S also provided its antiapoptotic effects on IRI of the heart and hepar via decreasing the caspase-3 expression [23, 24]. Additionally, H_2_S inflation during the warm ischemia phase showed better antiapoptotic effects indicated by less caspase-3 protein expression and TUNEL positive cells compared with ventilation in this study.

According to the results above, we found that lung inflation with H_2_S during the warm ischemia phase showed advanced protective effects on donor lung and IRI compared with H_2_S ventilation. The possible reasons existed. First, after the 1 h “hand-off” period, most donor lung alveolus was collapsed, and mechanical ventilation (MV) induced these alveolar periodic open and close which caused lung injury [25]. MV itself impaired alveolar epithelial and endothelial cells and increased pulmonary microvascular pressure and permeability of the alveolar capillary. Although protective ventilation strategies were operated in this study, it also led to significant expression change of lung gene which could be observed in the MV 90 min and increased lung injury [25]. Second, due to the gravity, disproportional expansion in the donor lung alveolus appeared during the MV. Even in the low tidal volume ventilation, it may also induce the excessive expansion of normal lung area to compress the closed lung area [26, 27]. Moreover, MV could cause the pulmonary surfactant change, such as reducing the adsorption capacity of phospholipids, increasing the conversion to SA from LA, and decreasing the LA activity [28], which were similar with the results in this study. These reasons all induced donor lung injury and serious inflammatory response [29] that was also showed in this study. Conversely, inflation characterized by static process provided a period of time allowing the gas to spread to the collapsed alveolus slowly which avoided the alveolus damage, improved the donor lung quality, and attenuated the IRI.

This study also has many limitations. In this study, the mechanism by which H_2_S affects surfactant is not studied, which needs to be explored in the future studies. Then, the changes of the exact components in the surfactant, like surfactant protein- (SP-) A, SP-B, SP-C, and SP-D, were not analyzed, which will affect the evaluation of the specific effects of H_2_S on pulmonary surfactant. Finally, the long-term effects of H_2_S on lung graft should be observed.

## 5. Conclusion

H_2_S inflation in vitro lung during the warm ischemia attenuated lung graft IRI and improved the lung graft function via improving the donor lung quality better by regulating the pulmonary surfactant compared with ventilation.

## Figures and Tables

**Table 1:** Experiment-related data (mean ± SD, *n* = 8).

Group	Number of rats excluded		Duration (min)		
	Donor	Recipient	Warm ischemia	Cold ischemia	Transplant operation
Sham group	—	0	—	—	—
Control group	0	1	191.7 ± 1.3	180.9 ± 0.5	15.8 ± 1.9
FS group	0	1	191.9 ± 1.7	180.6 ± 0.4	16.1 ± 1.6
VS group	0	0	192.0 ± 1.8	180.6 ± 0.5	14.3 ± 2.1

Rats in the sham group did not experience the warm and cold ischemia phase and were without transplantation. One recipient in the control group and 1 in the FS group were excluded because of the hemodynamic instability and the separation failure of the lung hilum. There was no statistic difference in duration in the control, FS, and VS groups. FS: H_2_S inflation; VS: H_2_S ventilation.

**Table 2:** The indices of blood gas analysis in recipients (mean ± SD, *n* = 8).

	Group	T0	T1	T2	T3	T4
PaO_2_/FiO_2_ (mm Hg)	Sham group	445 ± 18	442 ± 17	441 ± 14	446 ± 19	443 ± 19
	Control group	441 ± 15	424 ± 28	334 ± 30^∗^	288 ± 44^∗^	267 ± 51^∗^
	FS group	447 ± 19	440 ± 21	401 ± 31^∗^^#^	387 ± 27^∗^^#^	376 ± 27^∗^^#^
	VS group	448 ± 14	434 ± 33	384 ± 32^∗^^#^	352 ± 31^∗^^#△^	335 ± 36^∗^^#△^

pH value	Sham group	7.40 ± 0.01	7.40 ± 0.02	7.40 ± 0.01	7.39 ± 0.02	7.39 ± 0.01
	Control group	7.39 ± 0.01	7.39 ± 0.03	7.29 ± 0.15^∗^	7.22 ± 0.19^∗^	7.13 ± 0.16^∗^
	FS group	7.40 ± 0.01	7.39 ± 0.02	7.36 ± 0.09^#^	7.34 ± 0.10^#^	7.33 ± 0.07^∗^^#^
	VS group	7.39 ± 0.01	7.39 ± 0.01	7.34 ± 0.11^∗^^#^	7.30 ± 0.09^∗^^#^	7.28 ± 0.12^∗^^#△^

BE value (mmol/l)	Sham group	0.08 ± 0.03	0.07 ± 0.02	0.07 ± 0.03	0.06 ± 0.03	0.08 ± 0.03
	Control group	0.08 ± 0.02	0.07 ± 0.03	−2.99 ± 0.38^∗^	−4.37 ± 0.18^∗^	−4.56 ± 0.33^∗^
	FS group	0.08 ± 0.02	0.07 ± 0.03	−0.64 ± 0.30^∗^^#^	−1.12 ± 0.51^∗^^#^	−1.44 ± 0.30^∗^^#^
	VS group	0.07 ± 0.02	0.06 ± 0.04	−0.85 ± 0.30^∗^^#^	−1.37 ± 0.34^∗^^#^	−1.70 ± 0.25^∗^^#△^

PaCO_2_ (mm Hg)	Sham group	39.3 ± 2.6	39.1 ± 2.1	39.3 ± 1.6	37.9 ± 1.8	39.8 ± 2.4
	Control group	39.0 ± 2.2	39.0 ± 2.5	41.7 ± 2.8	38.8 ± 2.3	40.5 ± 3.2
	FS group	39.6 ± 1.9	38.5 ± 1.3	38.7 ± 2.4	39.2 ± 2.1	39.6 ± 2.7
	VS group	39.6 ± 2.9	39.3 ± 1.9	39.6 ± 2.5	39.1 ± 3.5	39.3 ± 2.2

T0-T4 represented the following time points: the baseline (3 min after ventilation), 3 min, 1 h, 2 h, and 3 h after reperfusion. PaO_2_/FiO_2_: partial pressure of arterial oxygen (PaO_2_)/fraction of inspired oxygen (FiO_2_); BE: base excess; PaCO_2_: arterial carbon dioxide tension. ^∗^*P* < 0.05 vs. sham group; ^#^*P* < 0.05 vs. control group; ^△^*P* < 0.05 vs. FS group; FS: H_2_S inflation; VS: H_2_S ventilation.

**Table 3:** The indices of blood gas analysis from pulmonary vein in recipients (mean ± SD, *n* = 8).

	PvO_2_/FiO_2_ (mmHg)	pH value	BE value (mmol/l)	PvCO_2_ (mm Hg)
Sham group	435 ± 19	7.40 ± 0.01	0.07 ± 0.03	39.4 ± 1.6
Control group	266 ± 46^∗^	7.13 ± 0.06^∗^	−4.41 ± 0.59^∗^	40.4 ± 1.9
FS group	383 ± 27^∗^^#^	7.36 ± 0.04^#^	−1.19 ± 0.31^∗^^#^	40.1 ± 2.6
VS group	341 ± 33^∗^^#△^	7.31 ± 0.06^∗^^#△^	−1.62 ± 0.38^∗^^#△^	39.9 ± 2.7

PvO_2_/FiO_2_: pulmonary venous oxygen tension (PvO_2_)/fraction of inspired oxygen (FiO_2_); BE: base excess. ^∗^*P* < 0.05 vs. sham group; ^#^*P* < 0.05 vs. control group; ^△^*P* < 0.05 vs. FS group; FS: H_2_S inflation; VS: H_2_S ventilation.

**Table 4:** W/D ratio and inflammatory mediators (mean ± SD, *n* = 8).

	W/D ratio	MPO (U/g)	IL-6 (pg/ml)	TNF-*α* (pg/ml)	IL-10 (pg/ml)
Sham group	4.5 ± 0.6	0.32 ± 0.12	118 ± 12	208 ± 20	234 ± 23
Control group	8.3 ± 1.4^∗^	1.66 ± 0.44^∗^	385 ± 53^∗^	537 ± 78^∗^	52 ± 27^∗^
FS group	5.0 ± 0.8^∗^^#^	0.51 ± 0.25^∗^^#^	162 ± 37^∗^^#^	249 ± 36^∗^^#^	202 ± 52^∗^^#^
VS group	5.7 ± 1.1^∗^^#^	0.85 ± 0.22^∗^^#△^	221 ± 28^∗^^#△^	300 ± 40^∗^^#△^	119 ± 36^∗^^#△^

W/D ratio: wet weight (W)/dry weight (D) ratio; MPO: myeloperoxidase; IL: interleukin; TNF-*α*: tumor necrosis factor-*α*. ^∗^*P* < 0.05 vs. sham group; ^#^*P* < 0.05 vs. control group; ^△^*P* < 0.05 vs. FS group; FS: H_2_S inflation; VS: H_2_S ventilation.

## Data Availability

The datasets used during the current study are available from the corresponding author on reasonable request.
